# Comparison of vaccination schedules for foot-and-mouth disease among cattle and sheep in Mongolia

**DOI:** 10.3389/fvets.2023.990043

**Published:** 2023-05-11

**Authors:** Gerelmaa Ulziibat, Eran Raizman, Amarsanaa Lkhagvasuren, Chris J. M. Bartels, Orgikhbayar Oyun-Erdene, Bodisaikhan Khishgee, Clare Browning, Donald P. King, Anna B. Ludi, Nicholas A. Lyons

**Affiliations:** ^1^Food and Agriculture Organization of the United Nations, Ulaanbaatar, Mongolia; ^2^Food and Agriculture Organization of the United Nations, Regional Office for Europe and Central Asia, Budapest, Hungary; ^3^Provincial Veterinary Service (PVS), Bayan-Undur soum, Orkhon, Mongolia; ^4^General Authority for Veterinary Services, Ulaanbaatar, Mongolia; ^5^The Pirbright Institute, Woking, United Kingdom

**Keywords:** vaccine, foot-and-mouth disease, vaccine schedules, immunogenicity, nomadic

## Abstract

Vaccines are a critical tool for the control strategy for foot-and-mouth disease (FMD) in Mongolia where sporadic outbreaks regularly occur. A two-dose primary vaccination course is recommended for most commercial vaccines though this can be logistically challenging to deliver among nomadic pastoralist systems which predominate in the country. Although there is evidence that very high potency vaccines can provide prolonged duration of immunity, this has not been demonstrated under field conditions using commercially available vaccines. This study compared neutralizing titres to a O/ME-SA/Panasia strain over a 6-month period following either a two-dose primary course or a single double-dose vaccination among Mongolian sheep and cattle using a 6.0 PD_50_ vaccine. Titers were not significantly different between groups except in sheep at six-months post vaccination when the single double-dose group had significantly lower titers. These results indicate the single double-dose regimen may be a cost-effective approach for vaccination campaigns supporting FMD control in Mongolia.

## 1. Introduction

Vaccines are extensively used in the control of foot-and-mouth disease (FMD), a disease of cloven-hooved livestock endemic through large parts of Africa and Asia. Mongolia is a vast, landlocked country with a long history of nomadic pastoralism (1). Herders dominate the rural economic landscape, depending on livestock production for their livelihoods. The low population density and nomadic lifestyle isolate many rural communities which can create problems for the delivery of veterinary services and vaccination (2). Regular incursions of FMD virus occur with a large impact among herders and cost to the government for control. In 2017, the cost of vaccination was estimated at approximately 60% of the total costs from reaction and expenditure and equivalent to US$4.3 million (3).

Post-vaccination monitoring is required to ensure vaccines are appropriate and effective (4). A previous study in Mongolia evaluated the immunogenicity of imported FMD vaccines in cattle, sheep and camels against high-risk strains for the region (5). This indicated that the current vaccines were well suited, although a two-dose primary course was required to avoid a rapid decrease in titers, and an oil-based adjuvant had a superior performance over an aqueous equivalent. A two-dose primary course is generally recommended for FMD vaccines, typically given 1 month apart followed by boosters every 4–6 months (6, 7). However, the extensive nature of the nomadic production system of Mongolian herders creates logistical difficulties in delivering a two-dose primary course frustrating disease control efforts.

A previous study under experimental conditions demonstrated that a single dose of a very high potency vaccine (>40 PD_50_) with an oil (Montanide^®^ ISA 25) adjuvant maintained high neutralizing titers over a 6-month period in sheep to the A22 Iraq vaccine strain (8). However, to the authors' knowledge this approach has not been used or evaluated under field conditions using a commercially available vaccine. This study aimed to compare the relative immunogenicities of a single injection of double the volume dose of a 6 PD_50_ vaccine with a conventional two-dose primary course among Mongolian cattle and sheep.

## 2. Methods

### 2.1. Study design

Studies were performed among cattle and sheep with eligible animals randomly selected from the farm for the study. Animals were assigned to one of three groups: single double-dose, two-dose and unvaccinated. The single double-dose (from here referred to as “double dose”) group were given a single injection of double the recommended volume of vaccine (2 ml in sheep, 4 ml in cattle). The two-dose group were given two single doses (1 ml in sheep, 2 ml in cattle) 14 days apart as per the manufacturer's recommendation. Unvaccinated controls received no intervention but were sampled on the same dates. Animals in each study were kept in the same group and had unique ear tag numbers to facilitate follow up vaccination and sampling. Serum samples were taken from all animals at first vaccination (0 dpv), 14 dpv, 56 dpv, 112 dpv, and 180 dpv with the unvaccinated controls sampled on the same day.

### 2.2. Farm and animal selection

Study herds were in Orkhon Aimag (Province), selected based on having no history of FMD, likely compliance with the study protocol, convenience in being close to Ulaanbaatar to facilitate repeat visits, and with no history of using FMD vaccine. Separate herds were used for the cattle and sheep studies. Animals were eligible for recruitment if between 4 and 18 months of age at the start of the study with no recent history of poor health. All animals were local Mongolian breeds. Before enrolment in the study, all animals were serologically negative to non-structural protein (NSP) antibodies. Animals were provided with ear tag identification at first vaccination with the first animals assigned to the two-dose group until the required number were reached, followed by assignment to the double dose group, then the controls.

### 2.3. Vaccine

The vaccine was commercially available (ARRIAH, Vladimir, Russia), contained strains from the O/ME-SA/PanAsia and A/ASIA/Sea-97 lineages, NSP purified, over 6 PD_50_ per dose, and adjuvanted with Montanide^®^ ISA 25. This was the same product as the previous study (5) but a different batch (number 120819, produced in August 2019) and delivered intramuscularly in the mid-cervical region.

### 2.4. Sampling and serology

Cattle and sheep were blood sampled through the caudal tail and jugular veins, respectively. Samples were kept on ice while transported to the laboratory where sera were separated and stored at −20°C prior to testing. All sera were tested for NSP antibodies using a commercially available ELISA kit (ID Screen^®^ FMD NSP Competition, ID Vet) at the State Central Veterinary Laboratory, Ulaanbaatar. No animals had received FMD vaccine previously, so NSP antibody negative animals were assumed negative for structural protein antibodies. Serum samples from 0 dpv were also tested using a serotype O solid-phase competitive ELISA (IZSLER, Brescia, Italy). Virus neutralization tests (VNT) were performed at the FAO World Reference Laboratory for FMD, Pirbright, UK as previously described (9). To reduce costs, VNT was only performed from 14 dpv onwards. Neutralizing titres were measured using the same field strain from the O/ME-SA/PanAsia lineage (O/MOG/13/2017) as the previous study (5), selected to provide a more conservative estimate than the previously used serotype A strain which was associated with higher titres.

### 2.5. Sample size calculation

The sample size was based on non-inferiority between the two protocols using previously reported titres at 56 dpv among sheep receiving a two-dose primary course (5). The non-inferiority margin was a 2-fold dilution, equivalent to log_10_0.3. Assuming a 5% loss to follow up, 32 animals were required (16 per group). Using data from the same study, the statistical power at 180dpv was 67%. The sample size was therefore inflated to 20 per group which was feasible for the selected farm and had a more acceptable power of 76%. Two unvaccinated controls were included as disease sentinels as recommended in the FAO-OIE guidelines for small-scale immunogenicity studies (4). All calculations were performed using the ssi module in Stata 14.2 (10). Although the sample size was possible in sheep, due to cost it was only possible to use half the number of cattle although due to the lower standard deviation the power at 180 dpv was acceptable at 76%.

### 2.6. Data analysis

Age and sex data were compared between groups using non-parametric Wilcoxan rank sum and Fisher exact tests respectively. VNT data were analyzed using multivariable interval regression, accounting for left and right censoring of neutralizing titres as described previously (5). Separate models were created for cattle and sheep, both including dosing group (two-dose vs. double-dose) and sampling time post vaccination as categorical variables. To estimate differences in titres at different sampling points, dpv was included as an interaction term in the model. Robust standard errors were estimated to allow for correlation of observations at the individual animal level. All analysis was done in Stata 14.2 (StataCorp LP, Texas, USA).

### 2.7. Ethical approval

Ethical approval for the study was granted through order 01/720 dated 15^th^ June 2020, of the Director in General Authority for Veterinary Services, Mongolia.

## 3. Results

Twenty-two cattle and 42 sheep were used for the studies. The first injection was administered on the 15^th^ June 2020 in both groups and species. In both the cattle and sheep double-dose groups, a single animal died during the study period at 137 and 173 dpv respectively. The reason for death in the cattle group was unknown and no post-mortem examination was performed whilst the sheep was predated by a wolf. One sheep in the two-dose group was successfully treated with parenteral antibiotics for an eye infection at 14 dpv and was retained in the study and given the second dose as per protocol. No local reactions at the injection sites were observed in any of the animals.

Cattle in the double-dose group tended to be older than the two-dose group. This was also the case in the sheep study, with the double-dose group also tending to have more females (Table 1). No animals showed clinical signs consistent with FMD during the study period and all samples were negative for NSP antibodies. Samples at 0 dpv were also negative to structural protein antibodies to serotype O using a solid phase competition ELISA (see Supplementary material).

**Table 1:** Descriptive data for animals randomly assigned to groups in a study (not including unvaccinated controls^a^), comparing two vaccination schedules for foot-and-mouth disease among cattle and sheep in Mongolia, 2020.

**Variable**	**Species**	**Category**	**Two-dose**	**Double-dose**	***P*-value**
Age (months)	Cattle	-	Mean: 12.0 Median: 12.0, Range: 11–13	Mean: 13.0 Median: 13.0 Range: 11–14	0.014
	Sheep	-	Mean: 13.1 Median: 14.0 Range: 12–14	Mean: 13.9 Median: 14.0 Range: 12–16	0.011
Sex	Cattle	Female	6 (54.6)	6 (54.6)	0.99
		Male	5 (45.4)	5 (45.4)	
	Sheep	Female	9 (45.0)	17 (85)	0.019
		Male	11 (55.0)	3 (15)	

For sex, column percentages are presented in parentheses. ^a^ Cattle: two controls were both 12 months of age, one female and one male. Sheep: two controls were both 14 months of age, one female and one male.

**Figure 1:**
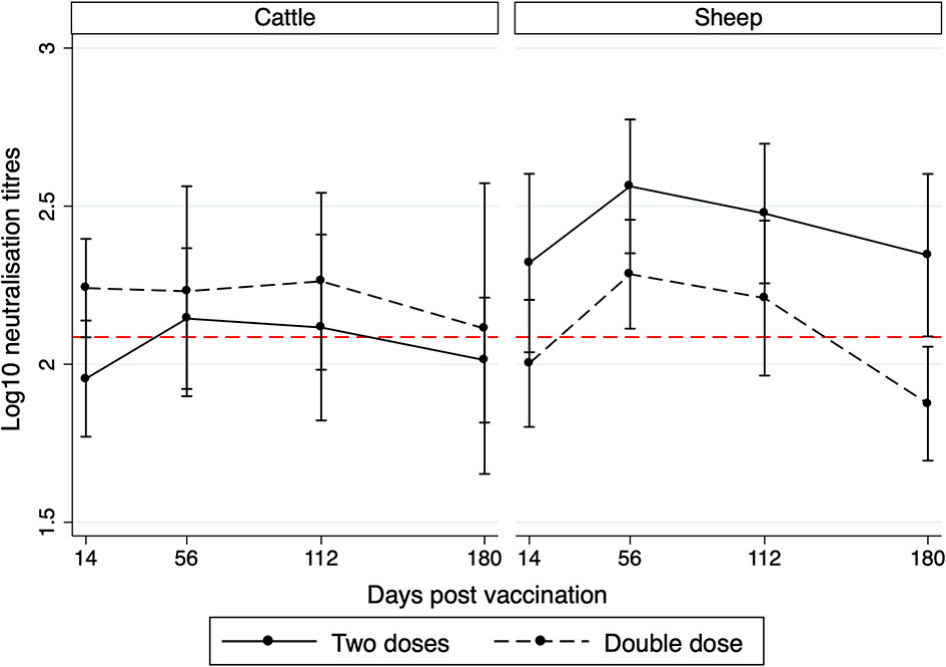
Post-vaccination neutralizing titers in cattle and sheep against O/ME-SA/PanAsia following either a two-dose primary course at days 0 and 14, or with a double dose administered at day 0. Sampling time was included in the model as an interaction term with dosing group, Mongolia, 2020. Data points represent model estimates ±95% CI. The horizontal dashed red line represents a titer that correlates with protection in 95% of cattle experimentally challenged with homologous strains from the serotype O lineage from the same laboratory (11). A similar estimate for sheep is not available in the published literature.

Amongst cattle, the neutralizing titres in the double-dose group were higher at all sampling points compared to the two-dose group, although there was no statistical evidence of a difference between groups (Table 2, Figure 1). A possible anamnestic response was observed in the two-dose group with higher titres observed at 56 compared to 14 dpv when the second dose was administered. Otherwise, titres were similar throughout the study period.

**Table 2:** Multivariable interval regression model comparing the impact of two foot-and-mouth disease vaccination schedules (double dose at day 0, and two dose primary course at days 0 and 14) and sampling time post vaccination on the titres against O/ME-SA/PanAsia, Mongolia, 2020.

**Variable**	**Category**	**Coefficient**	**SE (Robust)**	**95%CI**	***P*-value**
**Cattle**					
Vaccination schedule	Two-dose	Baseline	-	-	-
	Double dose	0.15	0.14	−0.13, 0.44	0.28
Sampling (days post vaccination)	14	Baseline	-	-	-
	56	0.090	0.098	−0.10, 0.28	0.36
	112	0.092	0.091	−0.087, 0.27	0.31
	180	−0.035	0.13	−0.030, 0.23	0.79
Constant	-	2.0	0.096	1.8, 2.2	<0.0001
**Sheep**					
Vaccination schedule	Two-dose	Baseline	-	-	-
	Double-dose	−0.33	0.13	−0.59, −0.079	0.010
Sampling (days post vaccination)	14	Baseline	-	-	-
	56	0.26	0.082	0.10, 0.43	0.002
	112	0.18	0.089	0.0080, 0.36	0.040
	180	−0.052	0.096	−0.24, 0.14	0.59
Constant	-	2.3	0.13	2.1, 2.6	<0.0001

CI, confidence interval.

In sheep, titres in the two-dose group were higher at all sampling points and the multivariable model indicated a significant difference between groups (Table 2). This effect was greatest at 180 days when titres for the double-dose group were significantly lower than the protective cut-off established in cattle (Figure 1). A possible anamnestic response was observed in both groups when comparing titres at 14 and 56 dpv (Figure 1).

## 4. Discussion

The results of this study indicated that neutralizing antibody titres to the O/ME-SA/Panasia strain after a single injection of a double-dose FMD vaccine were not significantly different to those elicited after a two-dose primary course delivered 14 days apart in cattle. In sheep, titres in the double-dose group were lower at 180 dpv and significantly below the protective cut-off established in cattle (11). There was some statistical evidence that ages and sex varied between the groups; however, the margin of difference was small and unlikely to invalidate the results of the study.

FMD vaccines are typically formulated into standard (3 PD_50_) and higher (6 PD_50_) potency types based on the number of 50% protective doses contained in each dose (12). The vaccine used in this study is advertised as being over 6 PD_50_, although the quantity of antigen is not stated. Very high potency (e.g., >10 PD_50_) vaccines have potential application for emergency reactive campaigns in an FMD-free setting due to the rapid onset of immunity supported by numerous studies under experimental conditions (13–15). However, consideration for longer duration of titres in a non-free setting has received less attention. One study quantified homologous neutralizing titres up to 6 months after a single dose of >40 PD_50_ vaccine in sheep (8). The results of that study indicated titres were maintained at “nearly peak” for up to 6 months in sheep with a Montanide^®^ ISA 206 oil adjuvant, although there was a gradual decline using the Montanide^®^ ISA 25 adjuvant as used in the current study.

The study was limited by not measuring titres to a strain from the serotype A lineage present in the vaccine due to limited resources. Serotype O was preferred since this is more commonly reported in Mongolia, and due to higher titres against the A strain in the previous study meaning that an O strain would likely provide a more conservative evaluation (5). Before any changes in vaccination policy are implemented, it would be prudent to measure the titres against a relevant serotype A strain.

Based on titres at 14 and 56 dpv, a possible anamnestic response to the second dose in the two-dose group was observed in cattle. This contrasts with sheep where there appeared to be increases between these sampling days in both groups. In the previous study using the same vaccine and neutralizing strains, no increase in titer was observed between day 14 and 56 after a single dose of vaccine in either species (5). “Late responders” in sheep have been reported previously with increases in titres occurring up to 3 months after a single dose of FMD vaccine adjuvanted with different oil adjuvants (16, 17). Such a prolonged response was not observed in the current study with an apparent decline at 112 dpv although there was no statistical evidence to support this observation and there were no samples taken at the 3-month timepoint to allow direct comparison.

In conclusion, these results indicate similar titres between groups of cattle given a double-dose or two-dose primary course of FMD vaccine over a 6 month period although in sheep the former was significantly lower at 180 dpv. Administering a double-dose may avoid the logistical difficulty of delivering a second dose to extensive and pastoralists production systems although further studies including an economic assessment comparing the two approaches in cattle and sheep would be worthwhile. Caution should be taken when extrapolating these results to other FMD vaccines with different potencies and strains for which bespoke studies are required.

## Data availability statement

The original contributions presented in the study are included in the article/Supplementary material, further inquiries can be directed to the corresponding author.

## Ethics statement

The animal study was reviewed and approved by the Director in General Authority for Veterinary Services, Mongolia. Written informed consent for participation was not obtained from the owners because this was obtained verbally with herders.

## Author contributions

GU contributed to the study design, led the fieldwork, and collected the data. ER conceived the study and contributed to the study design. DK and ALu contributed to the study design and interpretation of the results. CBa and ALk contributed to the analysis and interpretation of the results. BK and OO-E facilitated the field work. CBr led the laboratory work and contributed to the interpretation of the results. NL led the study design, performed the analysis and wrote the manuscript. All authors contributed to the article and approved the submitted version.
